# The Possibility of Eidetic Memory in a Patient Report of Epileptogenic Zone in Right Temporo-Parietal-Occipital Cortex

**DOI:** 10.3390/life13040956

**Published:** 2023-04-06

**Authors:** Brent M. Berry, Laura R. Miller, Meaghan Berns, Michal Kucewicz

**Affiliations:** 1 University of Minnesota Medical School, 420 Delaware St SE, Minneapolis, MN 55455, USA; 2 Neurology Department, University of Minnesota, 516 Delaware St SE, Minneapolis, MN 55455, USA; 3 VA Medical Center, 1 Veterans Drive, Minneapolis, MN 55417, USA; 4 Mayo Clinic, Department of Neurology, Department of Sleep Medicine, Department of Physiology & Biomedical Engineering, 200 First St. SW, Rochester, MN 55905, USA; 5 BioTechMed Center, Brain & Mind Electrophysiology Lab, Department of Multimedia Systems, Faculty of Electronics, Telecommunication and Informatics, Gdansk University of Technology, Gabriela Narutowicza 11/12, 80-233 Gdańsk, Poland

**Keywords:** refractory epilepsy, pair associative memory, eidetic, psychometrics, angular gyrus, dominant temporo-parietal-occipital junction

## Abstract

Eidetic memory has been reported in children and in patients with synesthesia but is otherwise thought to be a rare phenomenon. Presented herein is a patient with right-sided language dominance, as proven via multiple functional imaging and neuropsychometric methods, who has a seizure onset zone in the right temporo-parietal-occipital cortex. This patient’s medically refractory epilepsy and thus hyperactive cortex could possibly contribute to near eidetic ability with paired-associates learning tasks (in both short-term and long-term retention). There are reports of epilepsy negatively affecting memory, but as far as the authors are aware to date, there is limited evidence of any lesion enhancing cognitive functions (whether through direct lesion or via compensatory mechanism) that would be localized to a seizure onset zone in the dominant temporo-parietal-occipital junction.

## 1. Introduction

Eidetic memory, that being the capability to remember in near perfect detail a piece of information (or otherwise defined as a memory which is very vivid and has great potential for recall), is extremely rare. It is acknowledged that children possess more capacity for this than adults, suggesting that some developmental change disrupts the ability [1,2,3]. Scientists believe many people who retain these abilities into adulthood have better associative organization as opposed to true eidetic memory; many people displaying improved ability use memory tricks: for example, chess players can remember entire chess boards in game situations but if showed a randomly set board are no better than novices at remembering the layout [4,5]. It is not thought that children possess the associative context (defined as the ability to learn and remember the relationship between unrelated items such as the name of someone we have just met or the aroma of a particular perfume) but rather there may be an inherent quality in brain makeup which allows more efficacious encoding and recall of engrams. One theory is that there is a difference in the excitatory/inhibitory balance of neurons at that developmental stage [1,4,5]. Eidetic memory has been found in 2 to 10 percent of children aged 6 to 12 [6]. It has been hypothesized that language acquisition and verbal skills allow older children to think more abstractly and thus rely less on visual memory systems [6].

A few adults have had phenomenal memories (not necessarily of images), but their abilities are also unconnected with their intelligence levels and tend to be highly specialized [7]. In extreme cases, such as those of Solomon Shereshevsky and Kim Peek, memory skills can reportedly hinder social skills. Shereshevsky was a trained mnemonist, not an eidetic memorizer, and there are no studies that confirm whether Kim Peek had true eidetic memory [8]. That is to say the novelty of this report requires an understanding of the vagaries around the eidetic term itself, and the focus really is on what could be called enhanced cognitive capabilities in a specific domain or specific domains.

It is generally believed that patients with epilepsy, who also have in some sense a disorder of excitatory/inhibitory balance (over-excitation of a specific brain focus leading to seizures) have memory impairments rather than memory enhancement. Few counter examples exist to this; one possible case is that of Daniel Tammet in which researchers believed some type of ‘rewiring’ took place as a result of severe seizures allowing him synesthesia abilities which properly applied resulted in being able to memorize vast numerical sequences [9,10]. Here, we present another possible counter-example of a female with focal epilepsy in what is thought to be a hub for associative learning, whom possesses an eidetic paired associate learning ability. 

## 2. Clinical Analysis

A 27-year-old left-handed female patient was evaluated for better seizure control as part of phase 2 monitoring (in epilepsy evaluation, this means a patient has been deemed to have a focal lesion which might be amenable to surgical intervention, but instead of evaluating with scalp EEG—which is part of phase 1 evaluation—they generally require intracranial EEG evaluation). This patient did not have any history of perinatal problems, meningitis or encephalitis, or febrile seizures as an infant or child, and no family history of seizures. The patient did have a history of a fall from a bicycle at age 11 with a head injury without loss of consciousness but had about ten minutes of amnesia. The patient also had a history of depression, anxiety (for which patient was treated with citalopram and sertraline due to minimal interaction with anti-seizure medications), and bulimia, as well as osteoporosis.

The patient began having events at age 6. These involved staring and inability to respond for 15 to 20 s, although she thought that she did not entirely lose awareness. These occurred about five to six times per day. She had an MRI head scan which was reportedly normal, but an EEG was reportedly abnormal. Based on an abnormal EEG and the description of her events, she was started on carbamazepine, and she stopped having these events which were diagnosed by a primary care physician to be focal seizures with impaired awareness.

The patient after a period of seizure freedom elected with her prescribing physician to attempt to wean off the anti-seizure medication. This was in part due to side effects (patient had mild asymptomatic hyponatremia). Thus, at age 8, she stopped the carbamazepine but then had recurrent seizures of similar semiology as two years prior but at a frequency of up to 20 per day, so carbamazepine was restarted. After that, she had no further seizures for several years.

At age 13, the patient started having seizures which were somewhat different in that she would have a feeling as though she was tilting and losing her balance as well as staring, and then she would fall to the ground. In total, these events would last less than two minutes and would occur one to three times per week. There was not a clear life stressor associated with these events. There was no report of automatisms or focal motor semiology with these events, but she did state a feeling of loss of muscle tone in her legs. Her dose of carbamazepine was increased, and these episodes stopped after about a month, and she had no further episodes for two years.

At age 15, the patient had recurrent seizures with staring, sometimes with inability to respond and other times with preserved responsiveness. These were associated with a queasy feeling in her head and sometimes a feeling in her left side and would last about 30 s. At that time, she was started on oxcarbazepine in place of carbamazepine. However, she continued to have events. At age 20, she was switched to lamotrigine (200 mg twice daily), but she had increased event frequency of up to ten per day, so she went back to oxcarbazepine (600 mg twice daily). This resulted in a decreased seizure frequency, on occasion she had weeks without events but had no prolonged periods (>2 months) of seizure freedom. She had an outpatient EEG at this time, which was revealed by clinical note report ‘right temporo-parietal spike wave discharges seen rarely in sleep’. 

At age 23, her seizures of the same type (staring with or without loss of awareness and with or without epigastric sensation lasting 10–60 s) started occurring much more frequently (up to every day or multiple per day). An MRI head scan at that time was reportedly normal. An EEG at the time again showed right parietal-occpital sharp wave discharges in wake and sleep. Her dose of oxcarbazepine was increased up to 900 mg b.i.d., but she developed diplopia and dizziness, so the dose was reduced to 300 + 600 mg per day. On this, she had infrequent seizures at first, but over time, they once again became more frequent. Her dose of oxcarbazepine was increased to 600 mg b.i.d., but she continued to have seizures from a few times per week up to a few times per day. There was not any clear life stressor associated with the change in her seizures at this time.

**Figure 1:**
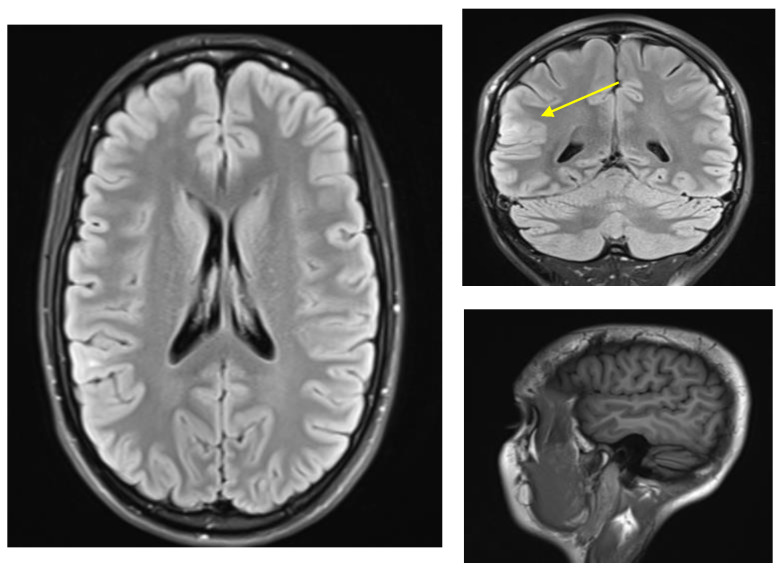
MRI-FLAIR findings of FCD in right posterior temporal-parietal-occipital junction. ((**Left**), axial, (**right top**) coronal, (**right bottom**) sagittal MPRAGE) Tiny focus of T2 signal abnormality involving the subcortical white matter of right superior temporal gyrus posteriorly (yellow arrow). Findings of this sort are suggestive of focal cortical dysplasia [11].

The patient was seen at our center at age 24. An EEG showed right posterior temporal polyspike discharges and sharp waves during wakefulness and sleep. An MRI head scan showed a focus of T2 signal abnormality involving the subcortical white matter of the right superior temporal gyrus posteriorly suggestive of focal cortical dysplasia (Figure 1). At that time, she was started on levetiracetam and eventually reached a dose of a 1000 mg b.i.d. while tapering off the oxcarbazepine. She was also started on clonazepam as needed for anxiety. She thought that the levetiracetam worsened her anxiety and she also had more frequent auras consisting of a feeling that her brain was “scrambled”. She therefore stopped the levetiracetam after a month and restarted oxcarbazepine 600 mg b.i.d. Soon after that, she was started on lamotrigine with the dose gradually increased to 100 mg b.i.d. Within a few weeks, she developed red spots on her hands and fingers, but this was not thought to be an allergic reaction. She was slowly tapered off oxcarbazepine over 2 months, at which time the red spots resolved. Given that the patient was on lamotrigine in the past, it was thought these spots were not related to lamotrigine. At age 25, the patient’s dose of lamotrigine was increased to 250 mg b.i.d. and later to 250 + 300 mg per day; however, she continued to have seizures (same as prior with staring +/− loss of awareness +/− epigastric sensation), and she also had side effects of diplopia and dizziness. Specifically, the patient stated she saw double when looking at objects in near and far distance, and this could be horizontal or vertical stacking. When she would close one eye, the double vision and the dizziness would resolve. For this reason, she was gradually tapered off lamotrigine and after that, she was on no antiepileptic medications and was having seizures up to three to four times per day.

The patient was seen here again at age 26. Blood tests including CBC with differential, chemistries, sensitive TSH, and magnesium were normal aside from a slightly low leukocyte count of 3.4 and neutrophil count of 1.66. Lamotrigine level was not detected. She was started on lacosamide with the dose gradually increased up to 100 mg b.i.d. On this, her seizures occurred less frequently, happening about once a day. However, she did note some problems with concentration. 

The patient underwent video/EEG monitoring. During this time, her dose of lacosamide was reduced to 50 mg b.i.d. The EEG showed right posterior and midtemporal (P8 and T8) spikes and sharp waves that were increased in sleep. A seizure was recorded showing the patient having eye fluttering and not responding, but she subsequently partially recalled the memory phase (please note testing at our institution follows a validated, standardized structured ictal-postictal testing battery similar to ILAE guidelines). Clinically, the event lasted approximately 25 s. There were no focal motor movements or automatisms during this time, and the patient did not clearly lose consciousness. Electrographically, there was a concurrent onset of a right temporal (maximal T10) semirhythmic theta discharge that spread to T8 and TP12 and lasted approximately 15 s. A second seizure showed the patient had very similar semiology to the first seizure with eye fluttering, no focal motor accompaniment, and no clear epigastric sensation, and by the time staff entered the room, the event was over, and she was able to read cards and follow commands. The patient reported that she had a seizure and that she had an unusual sensation in her head and down the left side of her body. Electrographically, there was a semi-rhythmic theta discharge in the right temporal head region, maximal at T10 but also seen at T8 and TP12. In a third seizure, the patient was in the bathroom when the nurse noticed that something was happening. The patient had an eye fluttering artifact on EEG. She reported an unusual sensation in her left arm and face. There was no clear EEG correlate to this event. A fourth seizure occurred with the patient asleep, and the electrographic onset occurred at the same time as clinical onset, with eye fluttering artifacts noted on the EEG (as evaluated by a trained epileptologist). The patient lifted her head off the bed, and it then turned in versive fashion to the right. She then had rhythmic non-voluntary jerking of the left greater than right leg and proceeded to flex the left hip. The patient then developed full body rhythmic movements consistent with generalized convulsions. The EEG showed a right temporal theta discharge that then spread and evolved, becoming a right frontotemporal theta-delta discharge before generalizing. At the end of the monitoring, the patient was put back on lacosamide 100 mg b.i.d. Her first three events were habitual, and the patient was not able to comment on whether her sleep event was habitual, but the patient had previously reported generalized seizures during periods of missing medications; all electrographic correlates did emanate from the MRI lesion suspect to be an FCD.

The patient had neuropsychological testing (Table 1). Neuropsychological testing was undertaken under normalized conditions by a trained psychologist with comparisons made to age-matched patients, and this revealed a reading level at the 83rd percentile. Arithmetic achievement was at a similar level. Verbal comprehension intellectual ability was at the 82nd percentile. Her perceptual–organizational intellectual ability was at the 91st percentile. She had previous psychological testing conducted when she was nearly 19 years of age. At that time, her verbal comprehension abilities were slightly higher. Her verbal abilities were somewhat higher and around the 99th percentile (verbal IQ measured at 162 where median is 100 std is 15; verbal IQ score is a measure of acquired knowledge, verbal reasoning, and attention to verbal materials). This decline in ability was thought to be associated with ongoing seizure activity and medication side effects at the time of secondary testing. 

Working memory was average. Performances on measures of cognitive speed and flexibility, however, were variable. Performance on one of these (Trails B) was mildly to moderately slow. Performance on the other (Stroop) was normal. Her untimed complex problem-solving ability or executive function was very good for age and educational attainment. Lexical and semantic fluency performances were normal, as was naming to confrontation. [12]

Her delayed recall for paragraph-length information was excellent. Her delayed recall for a supraspan word list was within acceptable limits, and she learned the word list with reasonable efficiency. Her delayed visual memory was normal as was immediate visual memory. Drawing of a complex geometric design was normal and well planned. However, on a batter of paired association, she reached high performance. The fact that the patient had displayed such abilities in her clinical neuropsychological evaluation prompted further testing by our group under a research protocol, which itself occurs while a patient at our institution is evaluated with phase 2 intracranial monitoring [13,14]. This research protocol instead of using short word lists used 300 paired associates. Interestingly, the patient’s recall was at 99% (298 out of 300). 

The task itself involved being asked to study a list of word pairs and then were later cued with one word from each pair, selected at random. The patient was then instructed to vocalize the partner of each cue word. Paired associate learning (PAL) tasks involve learning and memorizing the relations among stimuli that are not obviously associated. It is hypothesized that the brain needs this type of learning to support reading achievement by building on the associations between symbols and sounds independently from other language skills. However, other studies claim that the poor performance on PAL tasks of children with specific reading disabilities is more related to phonological deficits or the verbal demands of the PAL tasks than associative learning itself [15,16].

**Scheme 1:**
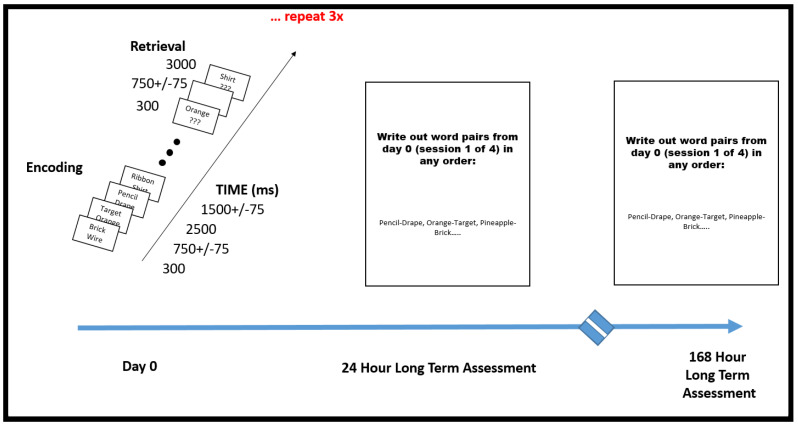
Paired Associate Learning Research Protocol. This additional testing was undertaken after it was demonstrated on neuropsychometric testing that the patient displayed superior abilities specific to paired associate learning. Researchers, after noticing remarkable performance, went off protocol and went beyond immediate short-term assessment to two more sessions of longer-term memory, asking the patient to simply write out the word pairs that she could remember from the immediate PAL session #1 (the first 300 word pairs). The patient was tested in this fashion 24 h later and then 168 h later. She was able to write out greater than 95% of the words in each session.

The encoding portion of PAL tasks can be visual stimulus–visual paired associate, verbal stimulus–verbal paired associate or cross-modal visual stimulus–verbal paired associate in nature [16,17]. This patient was tested with a cross-modal stimulus in that words are presented on a screen and recalled in an auditory fashion (see Table 1, Scheme 1) in a near identical fashion to that of Haque et al. [17]. 

Lists were composed of four pairs of short common English nouns, which were chosen at random and without replacement from a pool of 300 high-frequency nouns. Words were presented sequentially and appeared in capital letters at the center of the screen. Study word pairs were separated from their corresponding recall cue by a minimum lag of two study and/or test items. During the study period (encoding), each word pair was preceded by an orientation cue (a row of capital X letters) that appeared on the screen for 300 ms, which was followed by a blank interstimulus interval (ISI) of 750 ms with a jitter of 75 ms. Word pairs were then presented on the screen for 2500 ms, which were followed by a blank ISI of 1500 ms with a jitter of 75 ms. During the test period (retrieval), one randomly chosen word from each study pair was shown, and the participant was asked to recall the other word from the pair by vocalizing a response into a microphone. Each cue word was preceded by an orientation cue (a row of question marks) that appeared on the screen for 300 ms, which was followed by a blank ISI of 750 ms with a 75 ms jitter. Cue words were then presented on the screen for 4500 ms, which were followed by a blank ISI of 1500 ms. The patient could vocalize their response any time during the recall period after cue presentation. Vocalizations were recorded digitally and then scored manually for analysis. Responses were designated as correct, intrusions, or no response, when no vocalization was made or when the participant vocalized the word “pass.” Intrusion and pass trials were designated as incorrect trials. A single experimental session contained up to 25 lists [17].

**Scheme 2:**
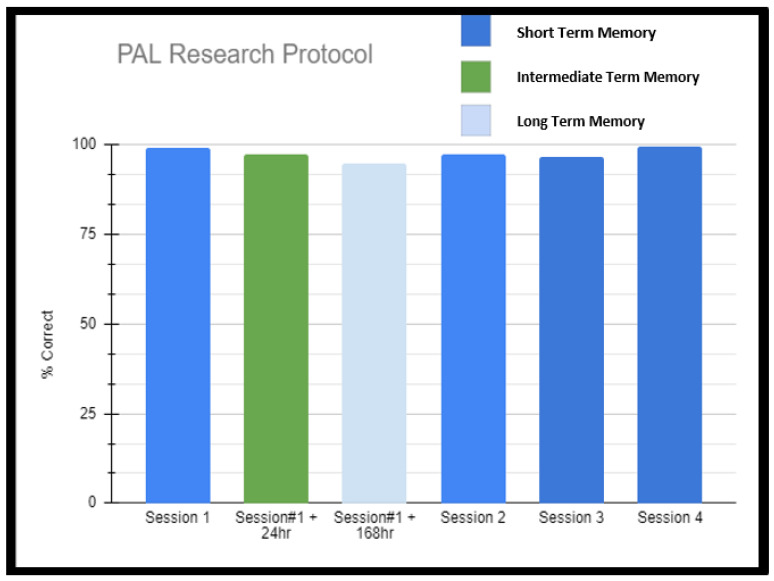
Paired Associate Learning Research Protocol Results. This additional testing was undertaken after it was demonstrated on neuropsychometric testing that the patient displayed superior abilities specific to paired associate learning. Researchers, after noticing remarkable performance, went off protocol and went beyond immediate short-term assessment to two more sessions of longer-term memory, asking the patient to simply write out the word pairs that she could remember from the immediate PAL session #1 (the first 300 word pairs). The patient was tested in this fashion 24 h later and then 168 h later. She was able to write out greater than 95% of the words in each session.

What was more interesting was that the patient was able to recite the paired words without the cue prompts (this was true for the vast majority of word pairs which the patient was able to recall with prompt). This was not a temporary or short-term memory phenomenon, as the patient was tested with similar length lists across the next 3 days and 7 days, obtaining results no less than 97% (Table 1, Scheme 2). The lists were randomly generated for each session using words from the same pool as the previous lists so that the memorization of default lists was not an issue. Extended testing beyond any of her neuropsychological clinical testing included PAL testing sessions four times (each made up of 12 word pairs encoded and then given a chance for retrieval repeated 25 times in what amounts to approximately a 45 min session). So, in total, the patient had four assessments of 300 word pairs each for 1200 word pairs on a single day. Researchers, after noticing remarkable performance, went off protocol and went beyond immediate short-term assessment to two more sessions of longer-term memory, asking the patient to simply write out the word pairs that she could remember from the immediate PAL session #1 (the first 300 word pairs). The patient was tested in this fashion 24 h later and then 168 h later. She was able to write out greater than 95% of the words in each session (see Table 1, Scheme 2). Given that this occurred inside the context of acute stress (surgery planning), the patient was also re-tested in a very similar fashion several months later after equilibration to her neurostimulator and had similar results (achieving greater than 97% on immediate assessment of massive word pair sets and greater than 95% on longer-term assessments involving writing out 300 words from one of the sessions on day 0).

The patient seemed not to have a full understanding that she had such an ability. In fact, she stated she had had trouble with school tasks, which required synthesis, even though she did well in certain classes where memorization may be helpful. Having difficulty with attention and synthesis (according to the patient’s teachers/professors and counselors), the patient was delayed from graduating college. 

**Figure 2:**
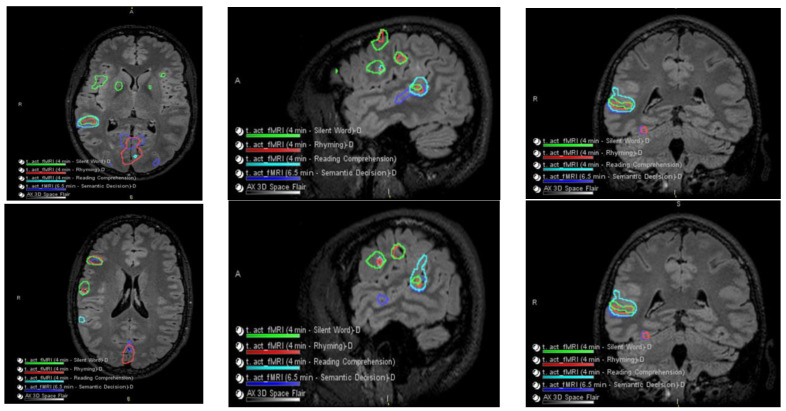
Language and Verbal Memory fMRI Testing. There were no obvious perfusion abnormalities. During cerebrovascular reactivity (breath hold), there was no cerebrovascular reactivity abnormality evident.

**Figure 3:**
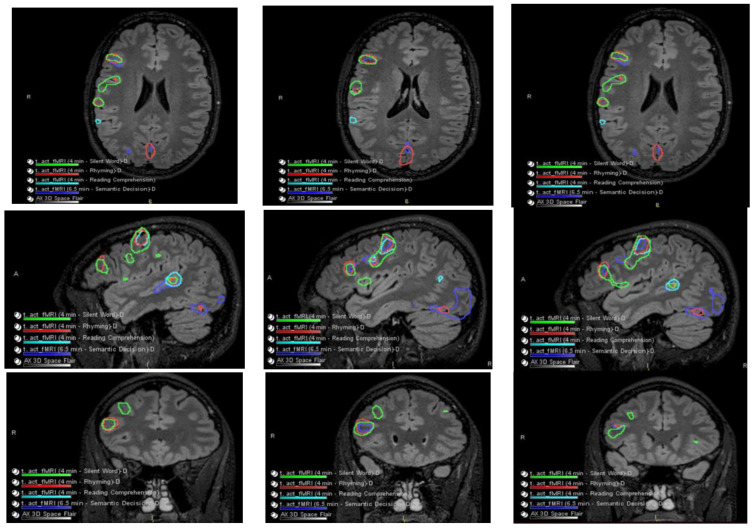
Co-localization of language-related activity with the silent word, rhyming, and reading comprehension tasks is also centered at the posterosuperior portion of the right pars triangularis. This is visualized on axial slides (ROW 1), sagittal slices (ROW 2), and coronal slices (ROW 3) suggestive of a right-sided Broca area. Other more posterosuperior right language-related activations are also seen with all 4 language tasks.

The patient had a functional MRI scan of the brain. This showed no change in the probable cortical dysplasia of the posterosuperior right temporal lobe at its junction with the angular gyrus. There was strong right cerebral hemispheric language lateralization. The right Wernicke area was just inferior to the presumed cortical dysplasia, although it was noted that language function could extend into the dysplastic cortical region with at least some language tasks (Figure 2 and Figure 3).

Language-related activation strongly lateralized to the right cerebral hemisphere with all four language tasks. The co-localization of activation centered at the posterior aspect of the right superior temporal sulcus with all four language tasks, which is suggestive of a right-sided Wernicke area (e.g., see overlaid summary maps axial sagittal coronal, respectively, in ROW 1).

With three of the four language tasks, areas of right Wernicke activation are very near each other in the right temporo-parietal-occipital cortex. These are within a centimeter, essentially abutting the inferior aspect of the adjacent presumed cortical dysplasia, although with the fourth (reading comprehension) task, language activation extends to/into the posterolateral aspect of the presumed cortical dysplasia (ROW 2). Of course, the volumes of these language-related activations as visualized on these maps are rather arbitrarily set by adjusting thresholds for sensitivity and specificity. 

No appreciable left cerebral language-related activation was seen with three of the four tasks. With the fourth task, i.e., the silent word generation task, there was a small amount of homologous left-sided Broca area activation and a small amount of left dorsolateral prefrontal cortex activation.

The patient’s findings were presented at an epilepsy surgical conference. It was the consensus of the conference that the patient would benefit from intracranial monitoring after implantation of a grid over the right mid and posterior temporal lobe around the area of focal cortical dysplasia. It was also suggested that a depth electrode should be put into the cortical dysplasia along with anterior temporal lobe strip electrodes.

**Figure 4:**
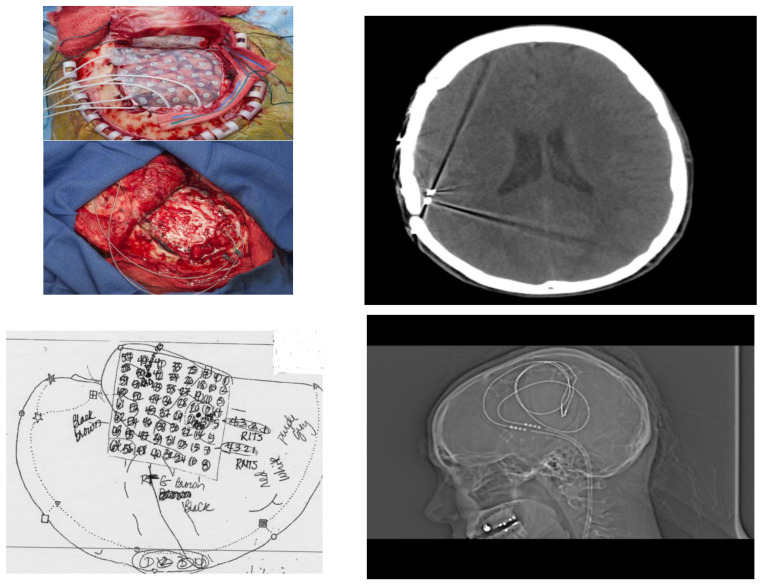
Operative Evaluation and Implantation of Neuromodulatory Technology. (**Left**, **top**) Patient underwent phase 2 intracranial electrode implantation with pre-planning (schematic **Left, bottom**). After identification of temporo-parietal-occipital junction seizure onset zone and with understanding from prior fMRI that this was eloquent cortex, neuromodulatory technology was implanted and visualized with CT scan and XR (**Right top**; **Right, bottom** respectively).

The patient had a stereotactic MRI for surgical planning purposes which again showed signal abnormality in the subcortical white matter of the posterior superior aspect of the right superior temporal gyrus near the junction with the superior marginal gyrus, most likely representing cortical dysplasia. The patient had implantation of intracranial electrodes in the right temporal region including an 8 × 8 right temporal grid and two four-contact right temporal depth electrodes as well as two four-contact right inferior temporal strip electrodes. She subsequently had video/EEG monitoring. This showed right posterior temporal spikes and sharp waves and numerous clinical seizures with onset in the right posterior temporal region at the junction of parietal and occipital cortex. She also had extra-operative cortical stimulation for functional cortical mapping. Eloquent cortex related to speech and language processing was mapped underneath the right temporal grid. These areas of the eloquent language cortex overlapped with her epileptogenic zone. For this reason, a trial of sub-threshold stimulation of the right temporal depth electrodes and the right temporal grid electrodes was carried out, which seemed to be beneficial in suppressing her interictal epileptiform discharges [18]. Consequently, she had removal of her intracranial electrodes and the placement of two right temporal depth electrodes and a brain stimulator. She did well following the surgery, achieving ILAE outcome 3 (Figure 4).

## 3. Discussion

In this patient’s situation, the paramount question is whether the enhanced abilities that were specific to a paired association memory are related to the patient’s seizure onset zone. Establishing which hemisphere is the dominant lobe (which was assumed to be the right hemisphere or at least co-dominant due to left handedness) is critical. Fortunately, language dominance testing at our facility involves rigorous assessments, including fMRI, which showed virtually all verbal faculties in the right hemisphere. Given this fact, the next question is in what way might an active seizure focus in an eloquent language area somehow be related to enhanced associative memory ability.

There is some evidence for a relationship between memory ability and GABA or glutamate measured by MRS, but it is not known how these relationships emerge during development [4,19,20,21,22]. It is not evident how the inhibitory and excitatory neurotransmission systems contribute to the overall network maturation, although there is more excitatory synapse/neuron weight (glutamatergic balance) in childhood compared to adulthood. [1,5]. Suffice it to say this glutamatergic component is likely dynamic in development [23,24], possibly as a result of changes in the way glutamatergic and GABAergic inputs interact with the intrinsic membrane properties of neurons [2]. While maintaining an appropriate balance between excitation and inhibition appears to be crucial for normal brain functioning, developmental changes in both glutamatergic and GABAergic mechanisms are thought to underlie the experience-dependent plasticity of cortical circuits and are deficient in a variety of neurodevelopmental disorders [3].

The finding that a particular brain region is associated with the acquisition of a cognitive skill at a specific developmental stage runs in line with neuroconstructivist theories of brain development. These theories surmise certain cortical networks emerge in the larger scheme of fixed brain architecture and are thus constrained [25]. For example, Kadosh et al. show certain brain regions are involved in face recognition, and these authors posit the glutamate/GABA ratios as assessed with MR spectroscopy are implicated with differential facial recognition performance between adults and children [7].

Another question is whether the particular brain region where the seizure zone lies in this patient is associated with paired associative learning. Vannest et al. show in fMRI studies that increased activation in dominant inferior/middle frontal gyrus occurs as well as in anterior cingulate, caudate, and temporo-parietal-occipital junction (the last of which was a bilateral phenomenon) [26]. Shafer et al. additionally show the importance of right temporo-parietal-occipital junction in associative learning [27]. According to Legaz in a study of neurodegenerative diseases, associative learning uses a broad fronto-insulo-temporal network but depends critically on temporo-parietal hubs and secondarily on fronto-limbic hubs, which are both related to context-target associative learning and social cognition [28]. Decety et al. believe paired association learning such as assessed by performance of the PAL test is dependent on the functional integrity of the temporo-parietal posterior junction as well as the entorhinal cortex, as has been evidenced in various lesion studies [29]. In their heavily cited work, Haque et al. also implicate the temporoparietal junction in verbal memory tasks [17]. 

Furthermore, it is important to consider GABA and glutamate in memory ability. Rossini et al. indicate a synaptic reorganization in the core of FCD, whereas no changes were reported in the perilesional tissue, as demonstrated by immunoreactivaties of vGLUT and vGAT expression [30,31]. In addition, there are studies showing genes mutations in the mTOR signaling cascade that regulates cell growth, proliferation, myelin formation and protein synthesis and are in association with neuronal hyperexcitability [32,33].

All of this taken together raises the possibility that the developmental changes in cortical neurochemical balance affect learning and the acquisition of new cognitive skills such as paired association learning. However, it also then raises the question of whether the excitatory/inhibitory balance, which is manifestly altered in epileptogenic tissue, could contribute to certain learning abilities. The limitations of this work are obviously that these are not reproducible results in many patients if any, and thus, much of the mechanistic explanation is conjecture. The strength of the study was the reproducibility within the patient, however, and of course its unique and exceptional role in the patient’s memory/learning abilities. These types of rare events can sometimes be as informative into brain functioning as larger cohort studies. It has been shown in multiple studies that epileptogenic discharges can disrupt memory formation [13]. Our report suggests a selective benefit for a memory function is related to the pathophysiology of epilepsy. 

## 4. Conclusions

Presented herein is a patient with right-sided language dominance as proven via multiple functional imaging and neuropsychometric methods, who has a seizure onset zone in the language dominant temporo-parietal-occipital cortex. Epilepsy is generally thought to contribute to negative effects on memory, but this patient’s medically refractory, epileptogenic cortex could possibly contribute to near eidetic ability with paired-associate learning tasks (in both short-term and long-term retention). This is a report raising the possibility of an ability-enhancing lesion in the dominant temporo-parietal-occipital junction, and while there are other reports that epilepsy may enhance memory ability, this would be the first report based off a focal epilepsy in this specific brain region. 

## Figures and Tables

**Table 1:** Neuropsychological assessment results along with expanded PAL testing diagram and results.

TEST	SCORE (Percentile)
Attention, Working Memory/Processing, Executive	
Trail making test part A	52
Trail making test part B	45
Stroop test part I	65
DKEFS Tower Test	91
Symbol Search WISC-IV	71
Language and Academic	
WISC-IV comprehension	82
WRAT-3 Reading	83
WRAT—arithmetic	79
Visuospatial	
Corsi test	65
WAIS block design	62
Rey complex figure	77
Learning and Memory	
Immediate recall verbal	78
Delayed recall supraspan	75
Immediate recall visual	68
Delayed recall visual	62
Paired Associates	
Immediate recall	99
Delayed Recall	99
Expanded Paired Associate Testing	No percentiles available
Repeating PAL testing research protocol 25 lists of 12 pairs	
Immediate assessment 4 sessions	298/300, 292/300, 290/300, 299/300
Long-term assessment 2 sessions (test the following day and then the following week based on immediate assessment #1)	24 h: 292/300 168 h: 285/300
Motor	Percentiles
WAIS digit symbol	22
Grooved peg board	
Right, left	32, 38
Beck Depression Inventory	8

## Data Availability

All the data supporting our findings is contained within the manuscript.
